# A Model for Dimerization of the SOX Group E Transcription Factor Family

**DOI:** 10.1371/journal.pone.0161432

**Published:** 2016-08-17

**Authors:** Sarah N. Ramsook, Joyce Ni, Shokofeh Shahangian, Ana Vakiloroayaei, Naveen Khan, Jamie J. Kwan, Logan W. Donaldson

**Affiliations:** Department of Biology, York University, Toronto, ON, Canada; Osaka University, JAPAN

## Abstract

Group E members of the SOX transcription factor family include SOX8, SOX9, and SOX10. Preceding the high mobility group (HMG) domain in each of these proteins is a thirty-eight amino acid region that supports the formation of dimers on promoters containing tandemly inverted sites. The purpose of this study was to obtain new structural insights into how the dimerization region functions with the HMG domain. From a mutagenic scan of the dimerization region, the most essential amino acids of the dimerization region were clustered on the hydrophobic face of a single, predicted amphipathic helix. Consistent with our hypothesis that the dimerization region directly contacts the HMG domain, a peptide corresponding to the dimerization region bound a preassembled HMG-DNA complex. Sequence conservation among Group E members served as a basis to identify two surface exposed amino acids in the HMG domain of SOX9 that were necessary for dimerization. These data were combined to make a molecular model that places the dimerization region of one SOX9 protein onto the HMG domain of another SOX9 protein situated at the opposing site of a tandem promoter. The model provides a detailed foundation for assessing the impact of mutations on SOX Group E transcription factors.

## Introduction

Upon the discovery of SRY (Sex-Determining Region Y), a transcription factor required for the expression of male sex-specific traits, a search for similar proteins revealed a large family of over thirty related transcription factors termed SOX (SRY-related HMG box). Sequence similarity among SRY/SOX proteins within the High Mobility Group (HMG) DNA binding domain typically exceeds 50%. Several structural and biophysical studies of the SOX4 [1,2], SOX5 [3] and SOX17 [4,5] HMG domains have shown that a considerable bend in DNA occurs when three-helix fold of the HMG domain and its peripheral basic regions interact with A/T rich sequences within the minor groove. Thus, SOX proteins not only act as transcription factors, they also act as architectural factors that influence partnerships with other proteins such as the POU homeodomain family [6,7]. Over the last five years, the SOX family has been extensively reviewed [8–10].

The Group E proteins SOX8, SOX9, and SOX10 are known for their ability to dimerize in a DNA-dependent manner [11–13] at sites bearing a (A/T)(A/T)CAA(A/T)G consensus sequence [14]. In contrast to Group D proteins (SOX5/SOX6/SOX13) that dimerize via a leucine zipper motif in a DNA-independent manner [15], the structure of the Group E dimerization region remains unknown. Mutations in the SOX9 dimerization region are associated with campomelic dysplasia, a syndrome associated with skeletal malformations, male-female sex reversal, and defects in the development of cartilage [11,16].

Early studies of the myelin glycoprotein Protein zero (P_0_) proximal promoter and collagen Col9a1 enhancer demonstrated that the dimerization region helps SOX Group E transcription factors function at a variety of tandemly inverted promoters that vary in binding site spacing and affinity [12,17]. These observations coalesced into the idea that there was a flexible coupling between the dimerization region and DNA binding domain [12,18]. A later promoter analysis of the miR-140 demonstrated that binding by SOX9 dimers and SOX5/6 dimers was necessary for full transcriptional activity [19]. While this result demonstrated a functional relationship among SOX family proteins, a SOX Group E protein cannot heterodimerize with a SOX protein from a different group [18]. Thus, Group D and Group E dimerization are represented by two different processes.

Here, we have performed a substitution mutagenesis study of the SOX9 dimerization region to define its sequence boundaries and its most functionally important amino acids. We have also identified two amino acids in the HMG domain that were essential for dimerization providing further evidence for the prevailing hypothesis that the dimerization region and HMG domain are directly coupled. To confirm this hypothesis, we demonstrated that a synthetic peptide derived from the dimerization region was able to bind preassembled HMG-DNA complexes. All of the experimental insights from this study were amalgamated into a high resolution molecular model.

## Materials and Methods

### Cloning and Mutagenesis

Portions of the human SOX9 gene (MGC14364; ATCC, Rockville, MD) encoding amino acids 110–184 (the HMG domain) and 71–184 (the dimerization region plus HMG domain) were amplified by PCR and inserted into the expression vector, pET15b (Novagen) via *NdeI* and *BamHI* restriction sites resulting in a 6xHis-tagged protein. A high solubility SOX Group E protein fragment retaining the ability to dimerize was manufactured by subcloning a synthetic gene block (GenScript) via *NdeI* and *XhoI* restrictions sites into the expression vector pET28 (Novagen) resulting in a 6xHis-tagged protein. The high solubility variant was used as a framework for making additional mutants in the dimerization region using the Quikchange method (Agilent). All constructs were confirmed by sequencing at the York University Core Facility.

### Protein Expression and Purification

Typically, 1–2 L cultures were grown at 25°C in LB media. Upon induction with 1 mM isopropylthiogalactoside at an A_600nm_ of 0.8, cultures were grown five hours further. As all expressed proteins were insoluble, the cell pellet was dissolved in a denaturing solution of TP300 (20 mM Tris-HCl + 50 mM sodium phosphate + 300 mM NaCl, pH 7.5) supplemented with 6 M urea and lysed further with a French press. The solubilized SOX9 protein was bound to a 10 mL nickel-NTA column (Qiagen) in the same buffer, successively washed with TP300 + 4 M urea, TP300 + 2 M urea, TP300 + 1 M urea and TP300 + 15 mM imidazole and eluted with ice cold TP300 + 20 mM EDTA + 1 mM phenylmethylsulfonylfluoride. After a brief concentration step, the protein was purified further by size exclusion chromatography using a Sephacryl S100 16/60 HiLoad column (GE Biosciences) equilibrated with 10 mM sodium phosphate buffer + 100 mM NaCl, pH 6.0. Protein concentrations were determined from their extinction coefficient at 280 nm as calculated by ProtParam [20].

### Electrophoretic mobility shift assay (EMSA)

An EMSA was used to directly visualize monomeric or dimeric binding of SOX9 proteins towards a 36 bp palindromic, oligonucleotide probe with two binding sites spaced 4 bp apart (CC36, GGGATCCTACACAAAGCCGGCTTTGTGTAGGATCCC) Annealing of CC36 was achieved by slow cooling a 100 μM solution in phosphate buffered saline from 95°C to room temperature. A binding reaction typically contained 2 μM oligonucleotide duplex and a 0.5–4 μM concentration of protein in a buffer of 10 mM sodium phosphate pH 6.0, 100 mM NaCl, 5 mM EDTA. After incubation on ice for 30 min, complexes were resolved using a 10% Tris-borate-EDTA gel. After soaking the gel for 15 min in a 1:10000 SYBR-Green-I solution (Invitrogen), visualization was performed using an Alpha Imager HP system (Alpha-Innotech).

### Peptide binding to preassembled SOX9 / DNA complexes

The previously described EMSA assay was used to determine if a peptide derived from the SOX9 dimerization region could bind HMG domain / CC36 DNA complexes in *trans*. The specific peptide used, termed D-peptide, was comprised of fluorescein isothiocyanate (FITC) fused amino terminally to a non-native glycine followed by amino acids 71–85 of the dimerization region (VSIREAVSQVLSGYD). This peptide was added to 1 μM stoichiometric SOX9 protein / CC36 DNA complexes and incubated on ice for 30 min. DNA-protein-peptide complexes were resolved using a 10% polyacrylamide gel containing 0.5x TBE buffer (45 mM Tris-borate, 1 mM EDTA) and visualized using a Bruker-Carestream 4000 MM Pro Image Station at wavelengths specific for FITC (*ex*: 495 nm, *em*: 519 nm).

### Estimation of binding affinities

An EMSA was also performed only radiolabeled oligonucleotides prepared by treatment with T4 polynucleotide kinase and ^32^P-γATP with a specific activity of 3000 Ci/mmol (Perkin Elmer). The specific oligonucleotide probes used were a CC36 double site described previously and a single site probe termed S9WT (GGGTTAACGAACAATGGAATCTGGTAGA). Each binding assay contained a constant 9 nM concentration of DNA, which was approximately one order of magnitude lower than the estimated *K*_*d*_ of the SOX9/DNA complex. Gels were imaged with a Typhoon 9400 imager (GE Life Sciences). Bands were integrated with GelEval v1.37 (FrogDance Software; Dundee, UK) and normalized to the observation from a highest protein concentration used in the titration. The data were fit to a four parameter logistic curve (A = [DNA]; B = [protein]; AB = normalized intensity of bands corresponding to protein-DNA complexes; *d* = maximum value, typically 1.0; *a* = minimum value, typically 0.0; *K*_*d*_ = mid-range of curve, *b* = steepness of curve, or Hill coefficient).

$$AB=d+\frac{a-d}{1+{\left(\frac{B}{K_{d}}\right)}^{b}}$$

### Molecular modeling

A molecular model of the dimeric SOX9 / DNA complex was assembled in six stages. All scripts and intermediate models used in these calculations are available at the York University Open Access Repository.

*Stage 1*: The SOX9 HMG domain sequence (101–184) was threaded into the crystal structure of SOX17/DNA complex (PDB:3F27; 80% identity to SOX9) with SWISS-MODEL [21]. Steric clashes were resolved and hydrogen atoms were added with CNS 1.3 [22] to make a model of one HMG domain bound to one binding site. After the molecular modeling was performed, a crystal structure of the monomeric SOX9 HMG domain bound to a single site was published in the Protein Data Bank (PDB:4EUW). The SOX17/DNA and SOX9/DNA structure were sufficiently similar to not warrant a recalculation of the entire model at this early stage (Fig A in S1 File).

*Stage 2*: A palindromic tandem site with 4 bp spacing was made using the software, 3DNA [23]. To maintain a bend and open minor groove, torsion angles from the protein/DNA complex in Stage 1 were used as input. From the resulting DNA structure, a set of intra- and inter-strand DNA-DNA distance restraints were calculated as well as a set of planarity restraints to help maintain base pairing.

*Stage 3*: Using the combined distance, angle and planarity restraints from the first two stages, a model of two SOX9 HMG domains bound to one tandem site DNA was calculated using a standard simulated annealing schedule in CNS akin to what is used in an NMR structure calculation.

*Stage 4*: The two SOX9 HMG domains from the model in Stage 3 were extended amino-terminally to include the dimerization region sequences as extended segments. A set of backbone torsion angle restraints and hydrogen bond distance restraints for helix α0 was synthesized using standard parameters for this secondary structure. Helix α0 was folded by subjecting the amino-terminally extended model to an additional round of simulated annealing.

*Stage 5*: At this stage, the dimerization helix α0 needed to be interfaced with the SOX9 HMG domain. Since the orientation of helix α0 on the HMG was not known, a study was performed separately on a simple system consisting only of helix α0 and α1/α2 helices of the HMG domain. Orientations of the dimerization helix α0 on the HMG domain were sampled by performing a rigid body docking simulation with HADDOCK v2.2 [24] and a set of short-distance ambiguous interaction restraints (AIRs) derived from mutagenesis experiments performed in this report. An AIR is a short (2.0 Å) distance restraint that can be fulfilled between any pair of amino acids in a specified set of possible interactions. AIRs can be generated using a utility on the HADDOCK website (*http://haddock.chem.uu.nl*). According the mutagenesis data, AIRs were created between I73, A76, V77, V80, L81 and Y84 in the dimerization region and A119 and L142 in the HMG domain. As recommended by the HADDOCK protocol, docking outcomes were improved by allowing the α0 helix to interact with a larger hydrophobic surface on the HMG domain. Thus, additional AIRs were made by amino acids near to A119 and L142 which were W115, L123, L130, T138, L139 and T146. From an ensemble of 400 trial structures, 20 lowest energy structures were chosen for assessment and clustered into three bins according to the orientation of α0 relative to α1 and α2. A candidate was the selected from the predominant bin and a set of fifty random distance restraints between helix α0 and the HMG domain were calculated.

*Stage 6*: All distance restraints from the previous stages were amalgamated and one final round of refinement with CNS was performed to produce a complete model of a dimeric SOX9-DNA complex. The linker region between the putative dimerization helix and HMG domain was left unrestrained throughout this stage.

## Results

### Expression of a high solubility SOX9 variant

**Fig 1:**
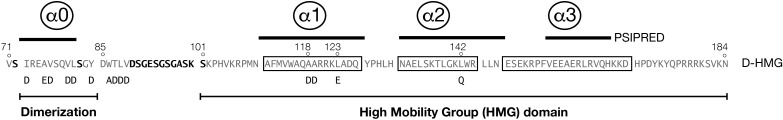
Human SOX9 variants. The sequence of D-HMG, a solubility enhanced variant of SOX9 incorporating the Group E specific dimerization region, a Gly-Ser rich linker in bold face, and the HMG domain. Two additional substitutions relative to the human SOX9 sequence (C72S, K82S) are also shown in bold face. Boxed sequences indicate the position of experimentally observed helices (α1/α2/α3) from a crystal structure of the SOX9-DNA complex (PDB: 4EUW) using the program, STRIDE [36]. Lines above the sequence indicate the position of helices predicted by PSIPRED [26]. Amino acids underneath the sequence indicate additional substitutions made to fine map the attributes of the dimerization region.

Initially, a protein fragment SOX9(71–184) was expressed to study dimerization. While this fragment was suitable for binding studies, it could not be concentrated to greater than 0.1 mM, thereby diminishing its suitability for future structural studies. Since a shorter SOX9(101–184) protein fragment spanning the HMG domain was soluble over a wide range of pH and ionic strength, we hypothesized that the solubility issues of the larger protein were due to several potentially solvent exposed hydrophobic amino acids in a linker region between the most essential amino terminal amino acids of the dimerization region and the HMG domain. We tested this hypothesis by expressing an optimized protein that incorporated the dimerization region from SOX10 (the only difference is that K82 in SOX9 is S82 in SOX10), and a new 12 aa. glycine/serine rich linker. One further substitution, C72S, was made to ensure that dimerization could be assessed in the absence of oxidation. Overall, these alterations resulted in a substantial increase in solubility (~0.8 mM was highest concentration tested). The sequence of this optimized, dimerizing protein fragment, termed SOX9 D-HMG, and several single amino acid substitution mutants made within that high solubility framework, are summarized in Fig 1.

### DNA binding affinity of dimerizing and non-dimerizing SOX proteins

**Fig 2:**
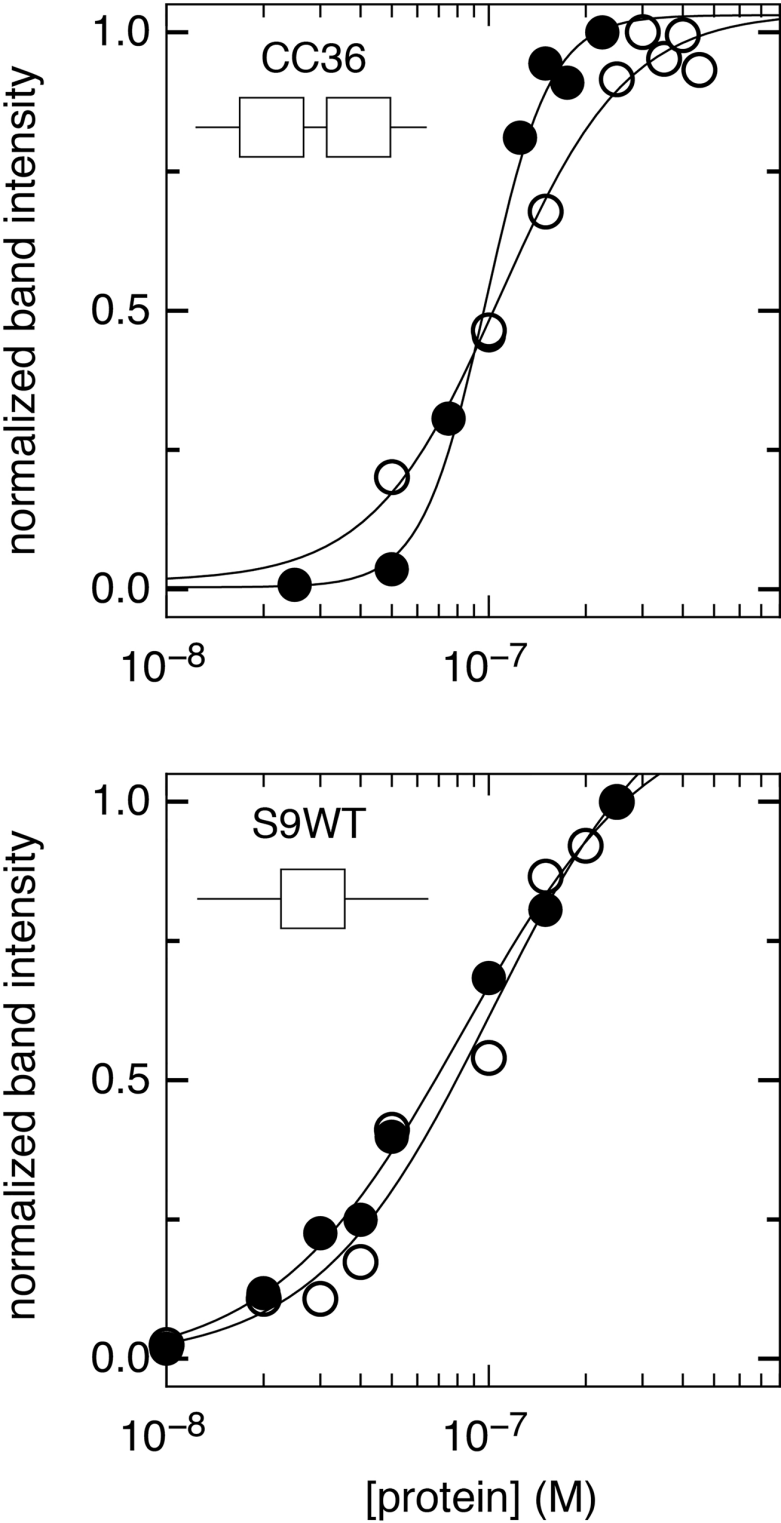
Binding of SOX9 proteins to single and double sites. **(a)** SOX9 protein fragments, either containing the HMG domain alone (HMG, open circles) or a high solubility variant containing the dimerization region and HMG domain (D-HMG, closed circles) were incubated at varying concentrations with ^32^P-labeled single site (S9WT) or double site (CC36) DNA and an EMSA was performed. Bands corresponding to single site and double site occupancy were measured, integrated and normalized. Each plot was fitted independently to a four parameter logistic curve.

An electrophoretic mobility shift assay (EMSA) was used to study the binding of SOX9 D-HMG and SOX9 HMG towards single site and double site DNA duplexes. The single site ^32^P-labelled probe was represented by S9WT, an engineered, high-affinity 29 bp sequence bearing one canonical AACAATG sequence [16]. The double site ^32^P-labelled probe was represented by CC36, a 36 bp palindromic sequence bearing two CACAAAG sequences from the stronger of two sites comprising the P_0_ proximal promoter [12]. An initial set of EMSAs were performed over a broad range of concentrations to estimate a *K*_*d*_. From that study, a refined range of protein concentrations from 10–450 nM was selected (Fig B in S1 File). Bands corresponding to the protein-DNA complexes were integrated, normalized to the highest concentration for presentation, and plotted as Fig 2. A four parameter logistic curve provided the best fit to the data for each condition from which affinities (*K*_*d*_) were calculated and presented in Table 1.

**Table 1:** Fitted parameters for the EMSA-based titrations in Fig 2.

SOX9 protein	DNA probe	K_*d*_ (nM)	Hill coefficient
D-HMG	CC36 (two sites)	98 ± 5	4.4 ± 0.6
D-HMG	S9WT (one site)	82 ± 19	1.5 ± 0.2
HMG	CC36 (two sites)	109 ± 10	2.2 ± 0.4
HMG	S9WT (one site)	101 ± 33	1.6 ± 0.5

Regardless of the protein (dimerizing or non-dimerizing) and DNA probe used (single- or double-site), the observed affinities were similar. This result may seem surprising since the dimerization region promotes the filling of double sites to such an extent that half-filled double sites are not observed. A closer examination of the plots in Fig 2 reveals one major difference; there is a steeper transition for the dimerizing SOX9 D-HMG protein on the CC36 double site than the non-dimerizing SOX9-HMG protein (Hill coefficients of 4.4 and 2.2, respectively) suggesting that more conformational changes are required to achieve binding of two dimerizing SOX proteins to double-site DNA.

Comparing the S9WT single-site CC36 double-site probes, affinity was favored slightly towards S9WT. This difference may reflect a preference for adenine (in S9WT) over cytosine (in CC36) at the first position of the binding site. Interpreting this result from another perspective, the SOX9 HMG domain is versatile enough to read out two different base pairs and still maintain a high affinity interaction.

As part of a comprehensive, early study on SOX Group E dimerization, a pulse-chase competition experiment was used to demonstrate that SOX10 D-HMG had a much longer residency on DNA than SOX10 HMG at the P_0_ proximal promoter C/C' tandem site [12]. From the decay plot presented, half-lives ~10 min and ~1 min were estimated for SOX10 D-HMG and HMG, respectively. According to the relationship, *k*_*off*_ = 0.693 / t_1/2_, the estimated half-lives are equivalent to off-rates of 1.6x10^-3^ s^-1^ and 1.6x10^-2^ s^-1^. Amalgamating these observations for SOX10 with the *K*_*d*_ values for SOX9 determined in this report, we estimate that the SOX9 D-HMG and SOX9 HMG proteins would have on-rates of 1.6x10^5^ M^-1^s^-1^ and 1.6x10^6^ M^-1^s^-1^, respectively. Thus, the available experimental evidence indicates that the dimerization region promotes both a slower on-rate and a slower off-rate relative to the HMG domain alone. The slower on-rate may be a consequence of two SOX Group E proteins requiring more time to scan DNA, find their respective binding sites, and come sufficiently close together to make new protein-protein contacts. Once two SOX Group E proteins are coupled to each other and to DNA, the slower observed off-rate is likely the consequence of the requirement for protein-protein and protein-DNA contacts to be severed before two Group E proteins can exit the binding site.

### Evidence for a possible amphipathic helix in the dimerization region

By replacing the amino acids 85–101 with an unrelated sequence to produce the SOX9 D-HMG variant used in this study, solubility was improved and the boundaries of the dimerization region were defined in accordance with earlier studies [12,25]. To determine what secondary structures may comprise the relatively short dimerization region, the human SOX9 sequence was submitted to PSIPRED [26] for analysis. Using the crystal structure of the SOX9-DNA complex (PDB:4EUW) as a basis for comparison, PSIPRED accurately predicted three helices (α1/α2/α3), although α3 did deviate from the crystal structure slightly in the length and position. This comparison is summarized above the sequence of SOX9 D-HMG in Fig 1. Most pertinent to this study, PSIPRED predicted that the dimerization region may be described in its entirety by one α-helix, spanning aa. 72–84 and designated as α0 throughout this report. The PSIPRED prediction is consistent with a previous circular dichroism study that demonstrated increased helical content in SOX9 D-HMG / DNA complexes versus SOX9 HMG / DNA complexes [18].

**Fig 3:**
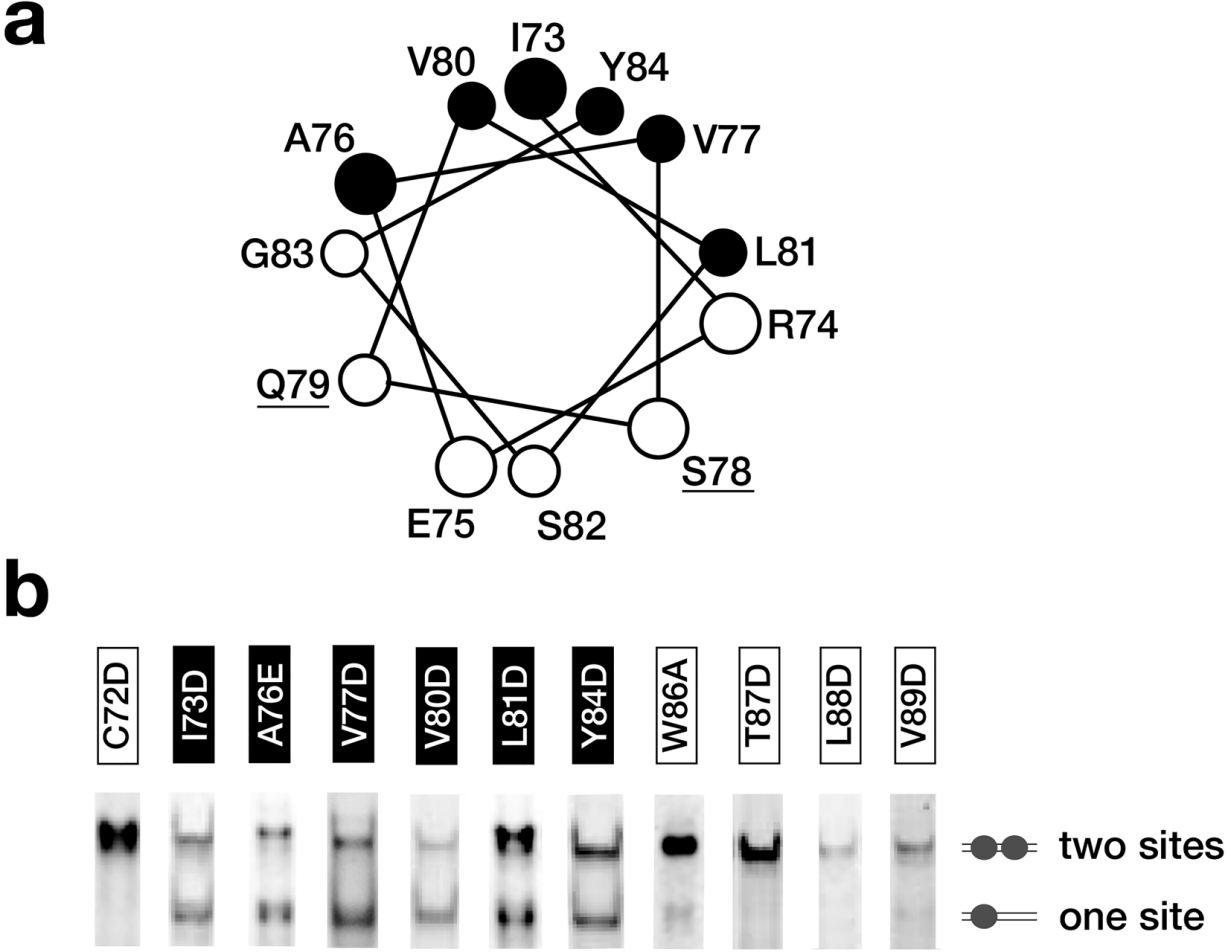
Nonradioactive EMSA assessment of substitution mutants in the dimerization region. **(a)** The dimerization region sequence was placed on a helical wheel with hydrophobic amino acids marked black and hydrophilic amino acids marked white to show the amphipathic nature of the predicted helix. S78 and Q79 (underlined) were substituted with alanine in a previous study with no effect on dimerization [25]. **(b)** For each substitution mutant presented in Fig 1, an EMSA was performed with a CC36 double site DNA probe and a substoichiometric protein to DNA ratio. Mutants with a nonfunctional dimerization domain are observed as a mixture of one site and two-site occupancies while mutants that retain a functional dimerization domain are observed exclusively as a two site occupancy. Mutants that do not have a functional dimerization domain from this qualitative assay coincide with the hydrophobic amino acids on the helical wheel.

A plot of the dimerization region on a helical wheel suggested a possible amphipathic helix with a hydrophobic face consisting of I73, A76, V77, V80, L81, and Y84 (Fig 3A). To assess the impact of these amino acids on dimerization, a substitution analysis was performed and analyzed by EMSA. The approach and visualization of the data is similar to a previous study [18], except that instead of using radiolabeled DNA, the same result was achieved by performing a titration with purified proteins at higher DNA concentrations and staining complexes directly with SYBR green. As shown in Fig 3B, sub-stoichiometric ratios of protein to DNA result in two bands for non-dimerizing mutants (two half-sites being partially populated) and one band for dimerizing mutants (two half-sites exclusively filled). From this visual assay, substitution of any of the six hydrophobic amino acids resulted in loss of dimerization on the double site probe. While the hydrophilic amino acids were not mutagenized in this study, S78A and Q79A substitutions have been noted previously to have no effect [25]. In summary, the body of mutagenesis data identify the importance of the hydrophobic amino acids as a platform for dimerization, possibly in the context of an amphipathic helix.

### Mutational analysis identifies sites within the SOX9 HMG domain required for dimerization

**Fig 4:**
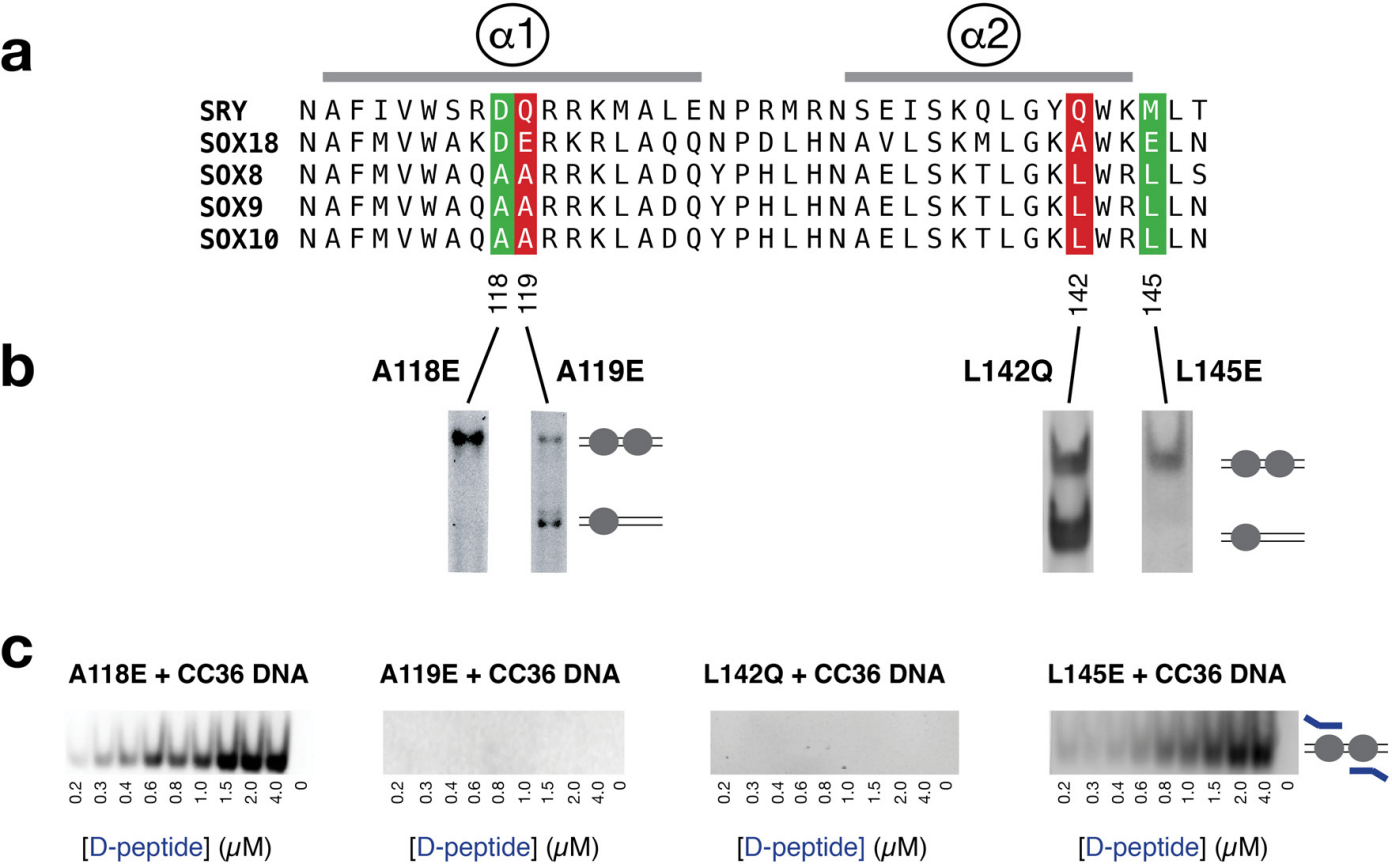
A peptide from the dimerization region binds preassembled SOX9 DNA complexes. **(a)** Sequence alignment of the HMG domains from three dimerizing Group E proteins (SOX8/9/10) and two non-dimerizing proteins, SRY and SOX18. **(b)** Four substitution mutants in the SOX9 HMG domain were chosen for analysis based upon sequence conservation among Group E family members and a high degree of side chain surface exposure. **(b)** Presentation of EMSA data is similar Fig 3. The observed single and double occupancy bands for the mutants A119E and L142Q demonstrated loss of dimerization (red). In contrast, the A118E and L145E mutants retain dimerization (green) **(c)** To solutions of stoichiometric, fully occupied complexes of protein (2 μM) and CC36 at (1 μM), an increasing amount of FITC-labeled peptide corresponding to amino acids 71–85 of the dimerization region (D-peptide) was added followed by an EMSA to resolve the complex. The presence of the peptide in the protein/DNA complex was determined by detection of FITC fluorescence (*ex*: 495 nm, *em*: 519 nm).

One important early insight into the role of the HMG domain in dimerization came from helix swapping experiments between SOX10 and a non-dimerizing Group C protein, SOX11 [25]. From this investigation, dimerization required contributions from helix α1 and helix α2 of the HMG domain. We hypothesized that the most important amino acids within α1/α2 of the HMG domain would be conserved among all Group E members and would be hydrophobic to complement the predicted amphipathic helix of the dimerization region. From a sequence comparison shown in Fig 4A, four substitution mutants within the D-HMG framework were assayed by non-radioactive EMSA. Mutants A118E and L145E had no effect on dimerization while mutants A119E and L142Q abolished dimerization (Fig 4B). Taken together, these mutants identify a potential hydrophobic platform for the dimerization region that is exclusive to SOX Group E proteins.

### Evidence for a possible interaction between the SOX9 dimerization region and the HMG domain

To determine if the dimerization region could directly interact with the HMG domain, a fluorescein tagged peptide (D-peptide) corresponding to amino acids 71–85 of SOX9 was used as a probe. From a titration of SOX9 HMG at concentrations up to 15 μM, no changes in fluorescence anisotropy were observed in a solution of 20 nM peptide indicating if there was a peptide-protein interaction, it was very weak (data not presented). This observation led us to hypothesize that the D-peptide may only interact with the HMG domain in preassembled HMG/DNA complexes since the HMG domain only folds completely in its DNA bound state [3,27]. To visualize a potential D-peptide-HMG-DNA ternary complex, fully occupied stoichiometric complexes of the four HMG domain mutants (A118E, A119, L142Q, L145E) with CC36 double site DNA were made and then successfully increasing amounts of D-peptide were added. The complexes were then resolved by EMSA and visualized for a fluorescein signal that was coincident with the D-HMG mutant complexes. As shown in Fig 4C, the D-peptide bound preassembled HMG/DNA complexes of the mutants A118E and L145E that retained the ability to dimerize, effectively competing away the endogenous dimerization region. Conversely, the A119E and L142Q mutants that lost the ability to dimerize could bind the D-peptide in *trans*. A analysis of the integrated fluorescent signals, demonstrated a linear relationship up to 2 μM, where a stoichiometric peptide-protein-DNA complex would be achieved. As a result, an affinity of the D-peptide for the for preassembled HMG-DNA complexes could not be determined.

### A molecular model of SOX9 dimerization

**Fig 5:**
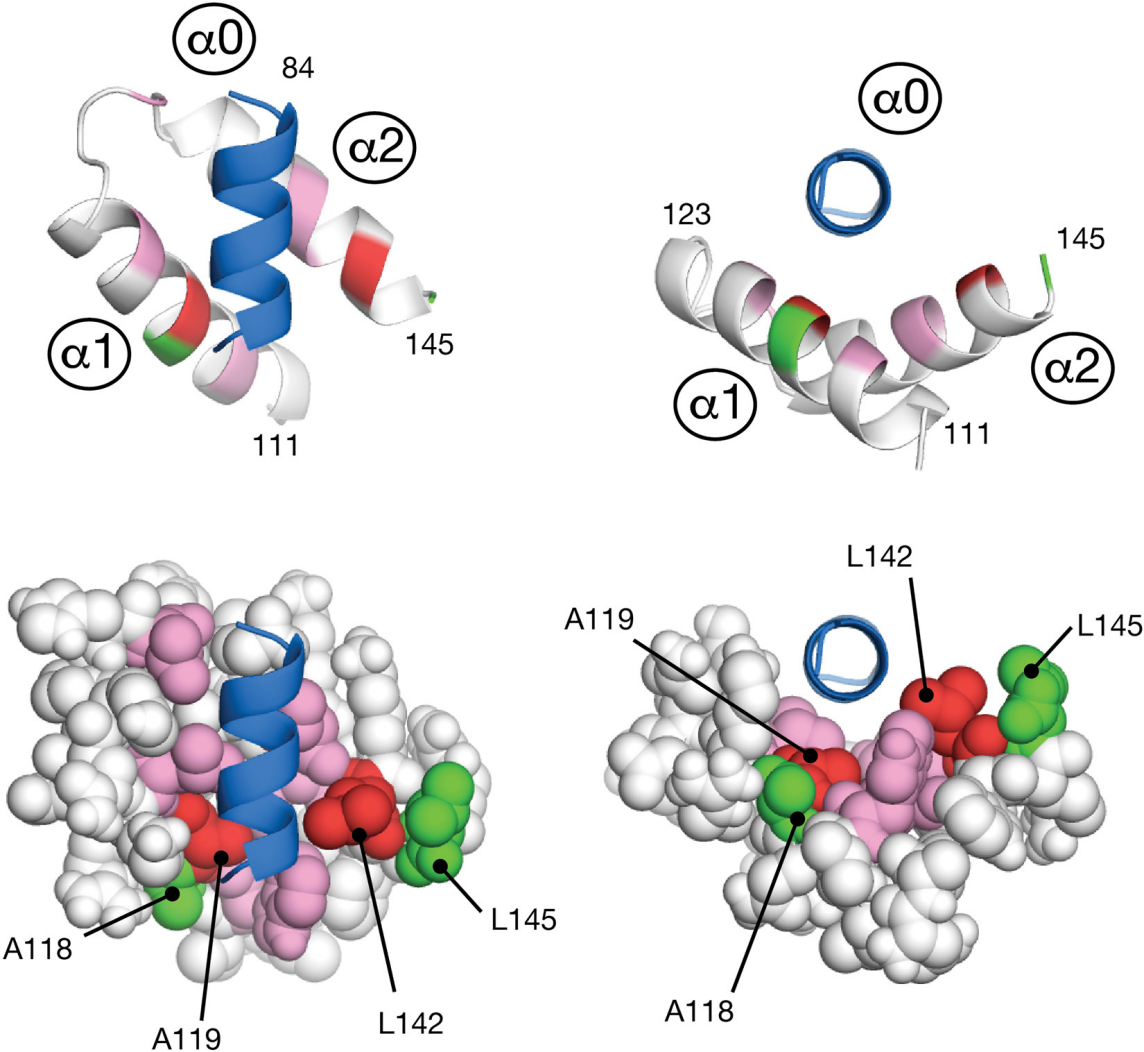
Molecular docking of a potential dimerization region helix upon the SOX9 HMG domain. Guided by substitution mutagenesis data, the dimerization region was modeled as one amphipathic helix (α0; blue), and docked onto a cleft formed by α1 and α2 of the HMG domain. Following the color scheme in Fig 4, amino acids that prevented and retained dimerization are red and green, respectively. Additional amino acids colored pink (W115, L123, L130, T138, L139, and T146) were include with A119 and L142 to form a contingous hydrophobic surface for peptide docking.

Towards obtaining the first high resolution view of the SOX Group E dimerization, the mutagenesis data was used as input in the form of ambiguous distance restraints for molecular docking experiment using the HADDOCK protocol [24]. To simplify the docking, only the dimerization helix (termed α0) and two helices of the HMG domain (α1/α2) were considered. A total of 400 trial models were initially made using rigid body dynamics to coarsely dock the dimerization helix α0 from a random starting position onto HMG helices α1/α2. The best twenty solutions from that stage that satisfied the experimental restraints were subjected to high temperature simulated annealing refinement. All twenty solutions are presented in Fig C of S1 File. Cluster analysis of the ensemble revealed seventeen solutions that placed the amino termini of the dimerization helix a0 and the HMG domain helix α1 in proximity. The remaining three solutions were in a secondary orientation that rotated helix α0 approximately 90˚ to place the carboxy termini of α0 and the HMG domain helix α2 in proximity. A search for similar three-helix topologies in the Protein Data Bank using SSM [28] revealed the crystal structures of the SIRV coat protein C-terminal domain [29] (PDB:3F2E; Cα RMSD 1.96 Å) for the predominant orientation and a Poly A Binding Protein (PABP) homolog [30] (PDB:1I2R; Cα RMSD 1.79 Å) for the secondary orientation. While either orientation of the dimerization helix α0 presented a plausible solution, the lowest energy model of the predominant α0 orientation was selected as the candidate for further stages of modeling because that α1/α2/α3 topology appeared to not require any additional structural contributions to make a complete fold. This candidate is presented in detail in Fig 5.

**Fig 6:**
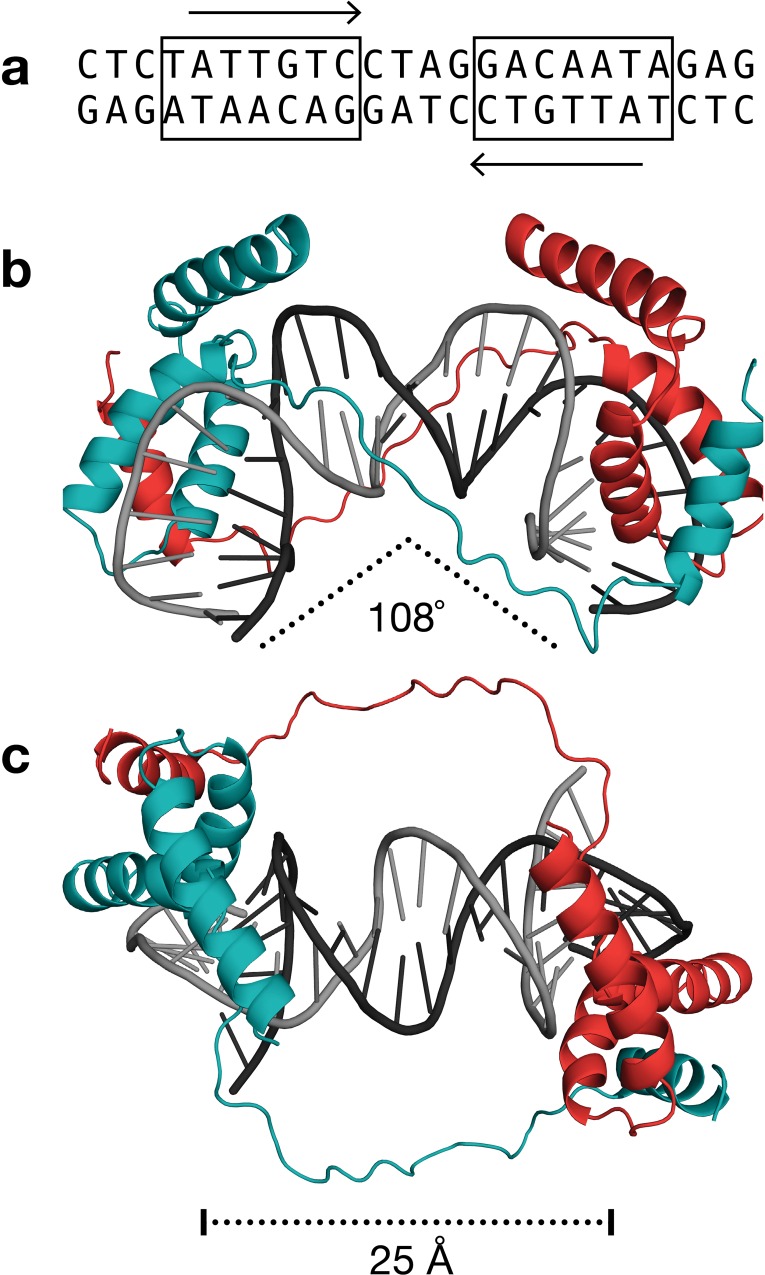
A molecular model of the dimeric SOX9 complex. **(a)** The sequence of the DNA duplex in the model. The two inverted sites in the palindrome represent canonical high affinity sequences for SOX family proteins. **(b,c)** Two views of the complex rotated 90° highlighting the bend in DNA and the distance in which the linker region can bridge the two proteins.

Building upon the results of the dimerization helix / HMG domain docking simulation, a complete molecular model of a dimeric SOX9-DNA complex was calculated using a combination of intramolecular protein-protein restraints to build an HMG domain and then duplicate it, intermolecular protein-DNA restraints to dock the HMG domains on tandem promoter, protein-protein restraints to dock the dimerization region on an opposing HMG domain, and DNA-DNA restraints to create a bend and open the minor groove (Fig 6). The resulting overall bend in the DNA, measured at 108°, was a consequence of the docking two HMG domains on a tandem site with no further adjustment. Since this value compares favorably to the 104° bend determined by an electrophoretic mobility study of SOX10 [25], the HMG domains and their DNA partners do not appear to require any further conformational changes to support dimerization. These leaves the dimerization event to be largely dictated by the interaction of the predicted dimerization helix α0 with the platform on the HMG domain that is formed upon DNA binding. The linker between the proposed dimerization helix and the HMG domain was modeled as being flexible, consistent with the PSIPRED secondary structure prediction and our observation that the linker could be replaced entirely with a segment of unrelated amino acids.

## Discussion

The observed affinity of SOX9 towards the single site and double site oligonucleotide probes used in this study is weaker by nearly two orders of magnitude than earlier reports for SOX4 (0.03 nM) and SOX5 (1 nM) [27,31]. This broad range of observed affinities among SOX proteins towards promoter sequences suggests that some may serve as better pioneer transcription factors than others for access to nucleosome bound DNA [32]. The weaker affinity observed for SOX9 may not necessarily be a detriment in this regard since transiently exposed DNA in nucleosomes tends to favor the binding of two or more proteins [33].

Similar dissociation constants were observed for dimerizing and non-dimerizing variants (substitution and deletion mutants) leading us to speculate that aside from creating a specific architecture through the binding of two proteins, the unseen consequences of dimerization may be to modify the kinetics of the interactions occurring at a given promoter or enhancer. Dimerization of SOX Group E proteins at tandem promoters may also promote the dimerization and activation of accessory factors that are coupled to them.

The mutagenesis based survey of the SOX9 dimerization region and HMG domain presented in this report builds upon several studies [11,17,18,25]. Combined, the body of available data suggests that the dimerization region consists of one amphipathic helix that binds to preassembled HMG-DNA complexes. In the absence of high resolution experimental data from NMR or X-ray methods, a molecular model was produced. From first inspection, the molecular model illustrates how a flexible linker between the proposed dimerization helix and HMG domain can permit a range of binding site intervals and bend angles [18]. While our observations for an A118E mutant demonstrated no loss of dimerization, an earlier study of SOX10 reported that a substitution equivalent to A118V in SOX9 was disruptive [25]. Our model provides a potential rationale for this observation as the glutamic acid side chain in the A118E substitution is able to point outwards into solution away from the dimerization helix α0 whereas a bulkier valine may create unfavorable steric clashes.

From the modeling of the dimerization helix α0 with the HMG domain, there remains the possibility that on a single site, helix a0 could bind helices α1/α2 of its HMG domain in the absence of a partner. Since the *K*_*d*_ of the SOX9 D-HMG and SOX HMG proteins are similar, it suggests that there is no major structural or kinetic consequence of self-binding. Thus, on single sites, SOX9 is free to function like any other non-dimerizing SOX family member.

The MADS domain transcription factor family also employs short secondary structure elements to link proteins already bound to DNA. At adjacent binding sites, a β-sheet in the MCM1 MADS domain is extended by a sequence donated by MATα2, an unrelated protein from the homeodomain family of DNA binding proteins [34]. MEF2 facilitates a different type of protein-protein interaction by offering a platform to dock an α-helix donated by the Cabin1 co-repressor [35]. In conclusion, we hope this study lays the foundation for the future high resolution characterization of multi-protein transcription factor complexes of SOX Group E proteins.

## Supporting Information

S1 File Fig A is comparison of the HMG / DNA molecular model used in this study with the crystal structure of the SOX9 HMG / DNA complex. Fig B presents the gel images used to determine protein-DNA binding affinities. Fig C is a workflow of the clustering and selection of a model describing the possible interaction of the dimerization region with the SOX9 HMG domain.
(PDF)
