# Supplementary material for: A Model for Dimerization of the SOX Group E Transcription Factor Family
Source: PLoS One. 2016 Aug 17;11(8):e0161432. doi: 10.1371/journal.pone.0161432 (PMC4988710; doi:10.1371/journal.pone.0161432)
Supplement: S1 File — Fig A is comparison of the HMG / DNA molecular model used in this study with the crystal structure of the SOX9 HMG / DNA complex. Fig B presents the gel images used to determine protein-DNA binding affinities. Fig C is a workflow of the clustering and selection of a model describing the possible interaction of the dimerization region with the SOX9 HMG domain. (PDF) [file pone.0161432.s001.pdf]

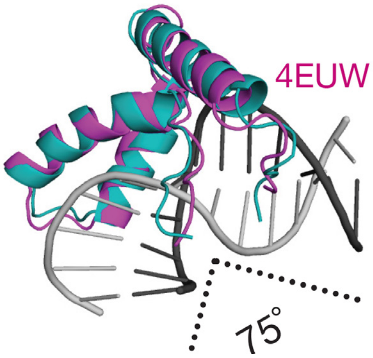

Fig A — Comparson of SOX9 HMG domain / DNA crystal structure and the dimerization model presented in this study. The SOX HMG domain / DNA structure (PDB:4EUW, purple) and one monomer from the dimeric model (teal) were superimposed according to their Cα atoms. The DNA bend angle is similar because similar restraints between the HMG domain and DNA were used in the molecular modeling. This comparison suggests that a platform formed between helix 1 and 2 of the HMG domain is sufficient, with no other conformational changes, to dock a proposed helix within the dimerization domain.

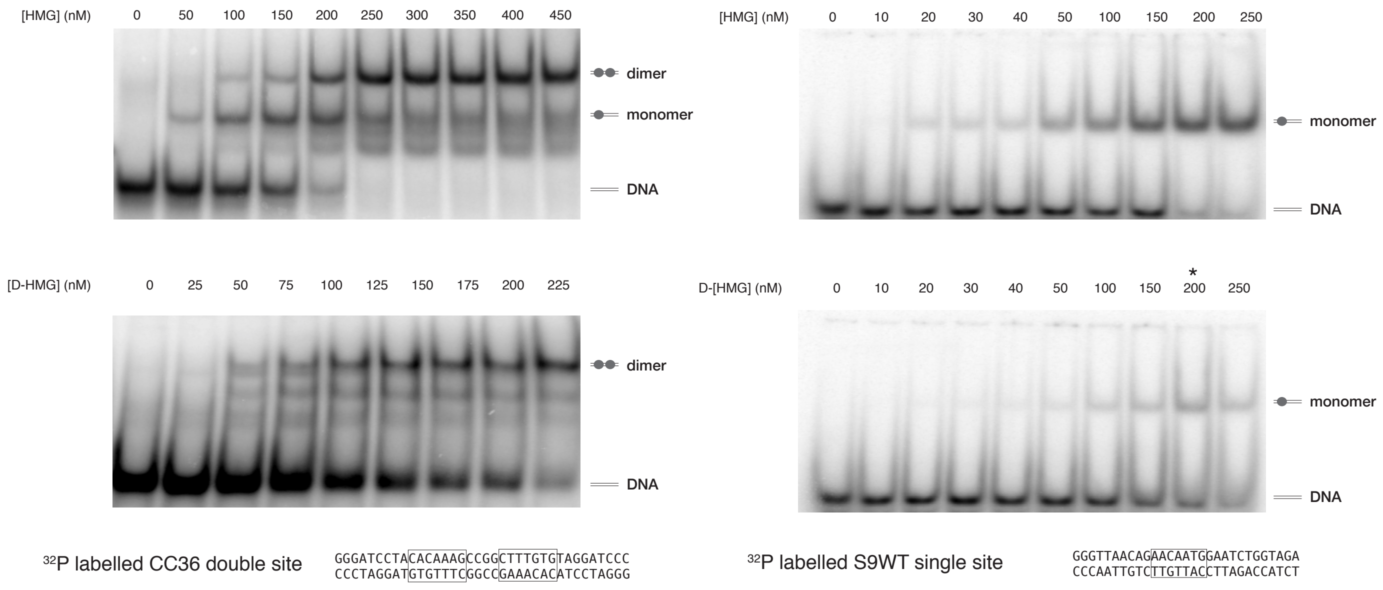

Fig B — Electrophoretic mobility shift assays (EMSA). A 9 nM solution of ^{32}P 5' labeled oligonucleotide containing either a inverted tandem binding site (CC36) or a single binding site (S9WT) was incubated for one hour on ice with varying concentrations of the SOX9 HMG domain (HMG) or a larger fragment that included the amino terminal dimerization region (D-HMG). The HMG/CC36 complex is interpreted as one HMG protein binding one of two equivalent sites in CC36. The D-HMG/CC36 complex is interpreted as a dimeric protein binding a dimeric site. As a result, in the CC36 titrations, the HMG protein concentration is expressed as a monomers and the D-HMG protein concentration is expressed as dimers. Complexes were resolved on a 10% polyacrylamide gel containing Tris-Borate-EDTA buffer and imaged with a Typhoon system (GE Biosciences). These bands were measured densitometrically with GelEval (FrogDance Software) and plotted as Fig 2 in the manuscript. An asterisk denotes lanes with anomalous band intensities.

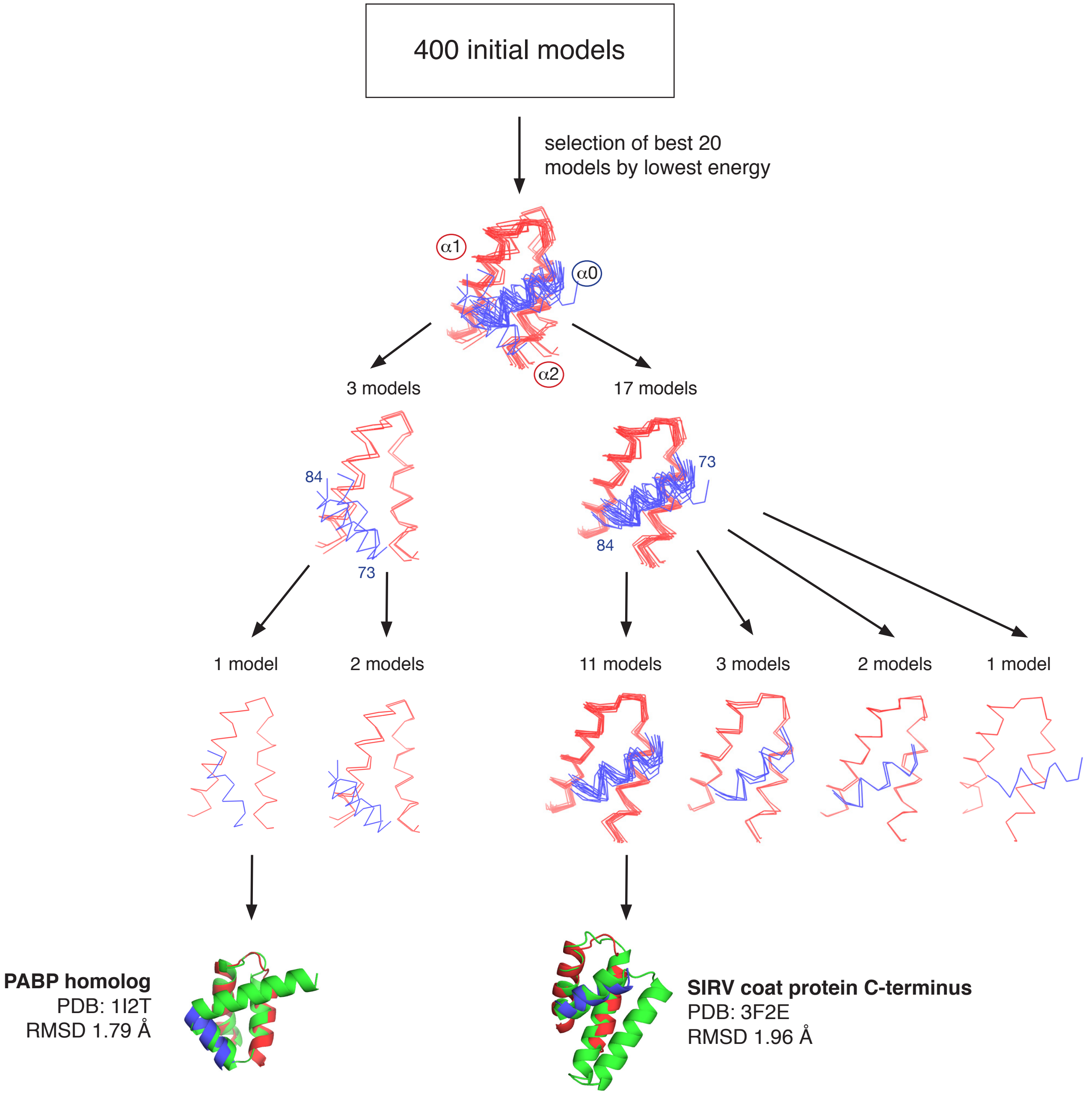

Fig C — Summary of HADDOCK driven docking of a possible dimerization helix α0 onto α1/α2 of the SOX9 HMG domain. A set of ambiguous restraints defining a surface on the HMG domain and dimerization helix were selected guided by mutagenesis data. Additional surface exposed amino acids were selected on the HMG domain to create a contiguous surface and improve docking. In the first iteration, the dimerization helix was moved to a random position away from the HMG domain and then a rigid body docking protocol was performed. From an ensemble of 400 models, the top twenty lowest energy models were selected for clustering and further analysis.Seventeen of the twenty models placed the dimerization helix in the same orientation, and in three remaining instances, was it rotated 90˚. A search for similar topologies in the Protein Data Bank revealed crystal structures of the SIRV coat protein C-terminus (PDB: 3F2E) for the predominant orientation and a Poly A Binding Protein (PABP) homology (PDB: 1I2T). The lowest energy model from one of the eleven predominant orientations was selected for further design of the dimeric SOX9 / DNA model.
